# Foreign

**Published:** 1840-12-19

**Authors:** 


### FOREIGN.

*Case of Phlebitis successfully treated.* By George Bell, M. D. &c.—Phlebitis is a disease over which local remedies have little control, and one whose general management does not appear to be accurately understood. Cases of successful treatment, therefore, are alike interesting and important, for it is from their multiplication alone, that correct principles can be deduced. In order to assist in the fulfilment of this object, we record the following history:

Richard Millar, aged 22, of spare habit, sallow complexion, and habitually temperate, was admitted into St. Bartholomew's Hospital on the 18th of June, 1836, labouring under œdematous erysipelas of the forearm, and simple integumental inflammation of the upper arm on the right side. His expression was anxious; the skin was hot and dry; the pulse frequent and full; the bowels were constipated; and the other symptoms which usually attend fever were observed.

He stated that, about ten days previous to his admission, he had been bled on account of a pain in his side, that on the following day he felt well, and returned to his work. The integument surrounding the wound made with the lancet became slightly inflamed and painful; but he continued at labour, heedless of the increasing pain and inflammation, until inclination and ability to be active had so diminished, that he was obliged to confine himself to the house. His symptoms gradually increased in severity, until the evening of the ninth day, when he presented himself at the hospital in the condition already described. He had not been subjected to any treatment.

Twenty-four leeches were applied to the upper arm; the limb was directed to be fomented; and a cathartic of calomel and the compound extract of colocynth was administered.

On the following day, the arm was less painful, but there was no abatement in the swelling, although the parts were less tense, and no improvement in the general symptoms. He now, for the first time, complained of pain shooting from the site of the wound towards the axilla; his intelligence was less acute, and his tongue was beginning to be incrusted at the edges. The basilic vein could be felt indurated nearly throughout its whole extent.

On the 20th, he was in the same state; the wound and the parts in the immediate vicinity were puffy, and obscure fluctuation could be felt. A small incision was made in the tumour, and about one drachm of purulent matter was evacuated. On introducing the probe, it was found that the instrument could pass in two directions only, viz. upwards and downwards, in the direction of the course of the vein, which led to the opinion that the matter had been evacuated from a venous abscess. This opinion was strengthened by the fact, that on gently pressing the vein from above downwards, an additional quantity of tinged purulent matter to the extent of two drachms escaped; and the vein, which previously felt hard and tense, had now become comparatively soft and pliant.

His pulse was still rapid, but compressible, and his bowels were inactive; the tongue foul; thirst moderate; and the skin dry but not parched.

A purgative enema was directed to be immediately administered; he was ordered to take every eighth hour a pill, consisting of two grains and a half of the chalk and mercury powder, and the same amount of Dover's powder, and to use the effervescing draught according to the symptoms.

21st. The constitutional symptoms were improved; the swelling and pain of the arm were abated; but he complained of pain below the short ribs on the left side, and acute pain in the left knee-joint. The pain of the side was relieved by pressure, but that of the knee was much aggravated by the slightest touch. The knee was fomented. From this time he gradually improved. In a few days the mouth was slightly affected by the mercury; the limb regained its normal condition, and the patient, as regards general symptoms, was convalescent. The vein was contracted to the size of a violin string. The alterative was omitted so soon as the mouth became affected, and some bitter infusion was prescribed.

On the 28th, he was quite convalescent, and discharged, with a recommendation to go to the country.

This case excited a good deal of interest at the time of its occurrence, and it is an important one in several respects. On the evening of admission it differed in no way from the common case of erysipelas with œdema, for the patient complained of nothing which could lead us to consider this lesion as secondary, either in importance or event. Our inquiries were directed towards the discovery of *phlebitis*, for considering the wound made by the lancet as the centre, starting point, or immediate cause of the erysipelas, we thought that it might give rise likewise to inflammation of the vein. This suspicion was not confirmed by aught seen or felt, for the redness and swelling of the upper arm effectually prevented any precise information being acquired by local examination. On the day succeeding that of his admission, matters were somewhat changed; there was pain distinctly referred to the course of the vein, the tenseness of the arm was diminished, and the indurated vein could be felt. Added to this, a tendency to typhoid fever was manifest. A question arose as to the propriety of continuing the antiphlogistic treatment. All the benefit to be expected from depletion or other depressing means had been already conferred, and we doubted the power of such measures, either to arrest or modify the progress of the local disease. Existing signs warranted this opinion, for the violence of the disease was abated, and the force of the constitution was declining; and the event of the following day confirmed it, for purulent matter had been already secreted, and was approaching the surface. We had to do, therefore, with the constitution; we had to deal with the fever which had been awakened.

There is a peculiarity in the fever which attends this and some other local inflammations which cannot be accounted for, either on the tissue affected, or by the intensity of the inflammation. It differs from the fever which accompanies pneumonia, pleurisy, and other simple acute inflammations, which is purely symptomatic, and it is identical with that described by the late M. Broussais, as symptomatic of *gastro enteritis*, or inflammation of the glandular and follicular tissue of the bowels. It has the character of what, in this country, is denominated continued fever; it is little tolerant of depletive measures, and but slightly affected by treatment of the local affection. It is essentially an idiopathic fever, excited by a particular cause, of which cause it immediately becomes independent. We hazard no theory regarding the primary cause of fever, but, having referred to the doctrine of Broussais, shall merely express a doubt whether the fever he recognizes as symptomatic of existing intestinal inflammation, is not rather dependent on disease having existed, by which the general irritability of the system has been so far modified as to render it obnoxious to common causes. In a word, we think that the fever is purely idiopathic in its nature; that it occurs, not because the gastro-intestinal mucous membrane is depraved, but because it has been depraved; and that erysipelas, phlebitis, &c., are merely exciting causes, acting on a system predisposed to fever, and waiting an opportunity to exhibit its predisposition.

Having hinted at what we consider to be the nature and origin of fever, it may be expected that something should be said about the nature of the causes of which it is so commonly regarded as symptomatic. A man receives a wound, lacerated, contused, or simple; erysipelas occurs, a degree of fever accompanies it; he is bled, and phlebitis ensues. Little benefit is derived from the depletion; the fever speedily loses any signs of vigour it may have at first exhibited; and it assumes a typhoid type altogether identieal with continued fever. This is a case which we have repeatedly witnessed. Here the man was predisposed to erysipelas; an acquired condition of the economy rendered him obnoxious to causes which, to the healthy, are sources of inconvenience rather than of danger. Anterior derangement was proved, by the changed irritability of the part in which the local disease appeared, and the constitution responded by a fever disproportioned in intensity to the exciting cause. Erysipelas is a local disease, recognizing a constitutional origin, and the fever excited by it is idiopathic in essence, originates from the same cause, and is born to immediate independence.

Reversing the order of events, local inflammation frequently arises during the progress of continued fever, and although the fever is its exciting cause, it is primarily dependent on the same anterior vicious working in the system which gave origin to the fever. The occurrence of inflammation in one tissue or organ rather than in another, can only be explained by the doctrine of peculiar susceptibilities. Inflammations occurring in this way are denominated spontaneous, by which term we understand that they arise independently of any influences external to the animal body. If this definition be correct, it implies some anterior change in the diseased capillaries, else when the same stimulus is circulating through the system, why does not general instead of local inflammation arise? It may or it may not be true, that fever is another name for general inflammation; but the question still returns, why in addition have we another and a distinct inflammation? One year a large proportion of the inhabitants of this town were affected with bilious diarrhœa; another year few escaped the depression of influenza; during a third year many above the average suffered the pains of rheumatism; and every year hundreds are seized with continued fever, to which is superadded a local inflammation, termed a complication, and occurring like an epidemic. Some peculiarity in the causes of the epidemic seems to determine the nature of the complication. The constitution has previously been so modified as to render one organ rather than another susceptible of generally acting causes. What may be affected through such a diluted imperceptible agent as these may be produced by the stronger and more sensible operation of unnatural, vicious, or civilized regimen, moral as well as physical. This seems to be the remote cause at once of the local disease and the fever it awakens, and as both are constitutional in origin, our remedies should be chiefly applied through the medium of the constitution. Experience shows, that topical remedies have little effect on the local evidences of constitutional ailment, and still less on the fever which the local disease kindles.

Erysipelas may be the exciting cause of phlebitis. A middle-aged man, of dissipated habits, received a wound on the hand; erysipelas ensued, which gradually extended up the arm to the body, so as to involve the shoulder and side of the chest. He died. On dissection it was found that the subcutaneous cellular tissue of the upper arm was affected, the limb was œdematous, the texture immediately surrounding the vein was infiltrated with purulent matter, the coats of the vein were thickened, the *vasa vasorum* were distinct, and the canal of the vessel was filled with purulent matter. We have reason to believe likewise that phlebitis is sometimes the result of burns. We have more than once made minute examination of the veins of subjects brought to the dissecting-room with marks of recent injury from fire. In these cases it was found that the plexus of veins at the bend of the arm, together with the main trunks, were effected in the manner above described. In addition to this there are other reasons for believing that burns sometimes prove fatal by inducing phlebitis. We have observed that burns by which the vitality of the skin is immediately destroyed, are, *cæteris paribus*, less fatal than those which excite such a degree of inflammation, as despite treatment, to advance to mortification or sloughing of that texture. The general symptoms in such cases are similar to those observed in phlebitis ; the fever is seldom of a character which demands decided antiphlogistic measures, but, on the contrary, is of such a nature as to require our being on the watch for the time when stimuli are necessary.

We have seen several cases in which the injury was confined to the upper part of the arm, and in which a fever, speedily assuming a typhoid type, set in, and carried off the patient. In comparing such cases with others, in which the vitality of the skin was destroyed by fire, and the patients recovered, we are inclined to believe that the constitutional symptoms were not, in the first class of cases, dependent solely on the integumental inflammation, but that some other local inflammation had arisen which determined the fatal event. The extent of the injury in the cases referred to, would not account for so serious a result. One fact, mentioned in the history of Millar's case, enables us to ask whether the practice of re-opening the wound in the vein, with the view to discover and evacuate purulent matter, might not with advantage become more general. We cannot see that it involves risk ; and it is reasonable to expect that it might frequently be productive of positive good.

With regard to the treatment of *phlebitis* in general, the practice pursued in the case of Millar seems to be worthy of imitation. It was adopted in his case, because we had frequently seen it successful in cases of erysipelas in which the constitution was similarly disturbed. Local depletion cautiously employed, alteratives, gentle laxatives, and enemata, succeeded by nutrients and a moderate use of stimulants, are the means best calculated to relieve the constitutional suffering, and conduct our patient safely through his convalescence. We say that local depletion should be cautiously employed, because danger may, and often does arise from acting on the imagination, that if it does no good it will do no harm, and we approve of alteratives, because we believe that the severity of the symptoms chiefly depends on a morbid condition of the intestinal and hepatic secretions, which can be most certainly remedied through their influence. We say this, not only because we have seen their beneficial effects in this case, but because we have witnessed their salutary operation in many cases of continued fever, evidently dependent on intestinal derangement; because we have observed their beneficial effects in cases of foul ulcers, and ill-conditioned wounds in which the general symptoms were similar to, though not so intense as those which accompany *phlebitis*; and because we have found them to be valuable agents in many cases where long-continued bad health was clearly referable to a depraved condition of the gastro-intestinal mucous membrane, as indicated by the tongue, skin, eye, temper, and the mental and corporal activity of the person. This would be a fitting place to enlarge on sympathy with a view to explain all this. We might talk at length and much more mysteriously about the sympathy which exists between the stomach and other parts of the system. We might quote the numerous propositions of authors, criticise their logic, and express wonder at their powers of supposition. We might consider their data, modify their reasoning, and draw new, perhaps opposite conclusions ; but in so doing we should be aiding and abetting in mystifying what has been already sufficiently obscured. Thus, although a fresh hypothesis might be invented, and words explanatory thereof traced, still no one would be benefited thereby ; for it is absurd to speculate on conceptions of whose genuineness we have no certain tests. Too much caution cannot be observed in the application of the inductive method of reasoning to the solution of questions connected with a purely practical science.

In the cases alluded to, we dread the active employment of cathartics, unless the bowels are distended with feculent matter, and a necessity evidently exists for their immediate evacuation. In such instances we should employ purgatives with a view merely to unload the bowels, and not with that perseverance which implies a belief in their power to remedy the lesion which determined the lodgement. We here desire that our opinion be not regarded as opposed to the doctrine so ably propounded by the late Dr. Hamilton, whose name is so closely and justly associated with the principle of employing laxatives without purging. Dr. Hamilton refers to cases in which it is evident that the symptoms are owing to abdominal irritation produced by the accumulation of feculent matter; and not to cases where they are dependent on a depraved state of the gastro-intestinal mucous membrane, of which the lodgement of fæces is not the most serious effect, or that to which the physician should primarily and principally direct his attention. We employ purgatives in such cases merely to unload the intestines, to remove accumulated secretions, and not with any intention to remedy the lesion which exists, which is more certainly rectified by the judicious employment of mercurial alteratives.

We have frequently observed the co-existence of erysipelas, phlebitis, and other local morbid states, with abdominal derangement ; the latter having been known to exist prior to the former, or the fever which accompanies them, and, therefore, independent of both. The frequency of the coincidence awakens a belief in their connection ; and the result of treatment directed towards remedying the abdominal lesion justifies the opinion, that that connection is relationship. We have spoken of alteratives as a class of remedies on whose power to rectify the abdominal derangement we place considerable reliance; and we are the more encouraged to do so, because we have generally observed that slight affection of the gums, or fœtor of the breath, and the commencement of a return towards health, are simultaneous events. Although not quite relevant to the subject, we may be allowed to remark, that, in the treatment of delirium tremens by calomel and opium, abatement of the symptoms has almost uniformly been coincident with the characteristic effects of the mercurial. This has been so much the case, that, to us, it appears a good fact on which to ground our prognosis, however slight the change may be. It is an evidence that the constitution is still susceptible of the influence of remedies.

Derangement of the biliary organ is frequently associated with the lesion of the stomach and bowels referred to. Whether it be affected primarily or secondarily, the result is the same; for once established, the two diseases react on each other, and the discord is kept up. Each lesion becomes a strong reason for the persistence of the other, and our object is to remove one, for thereby we shall more readily silence the other. We do not pretend to know the cause of the primary lesion, for it may or it may not depend on the causes to which it is commonly attributed; but of this we are assured, that the diseases may go on, though the primary cause has long ceased to operate, for two lesions exist; they are springs, and the pendulum which vibrates between them, striking alternately against each, is sent back with almost undiminished force. But vital strength will yield to continued calls for its exertion; by degrees this energy will diminish; by degrees this reciprocal action will fade; in time it will entirely cease, and the patient be a miserable dyspeptic. The two lesions are now quite independent of each other, and for their removal a doubtful and experimental and complicated treatment is required.—*Edinburgh Medical and Surgical Journal.*

---

*Cases of Scarlatina, with Observations on the Nature and Treatment of that Disease.* By William Cullen, M. D., Cupar-Fife.—During the whole of the winter of 1838–9, scarlet fever prevailed in this town and neighbourhood; and the district to the southeast between this place and the coast, has been visited with it very generally. Its diffusion has been slow, but this is one of the most curious circumstances of the disease. A great proportion of the cases have terminated fatally, which is my chief reason for writing on the subject. In the village of Kilconquhar nine have died out of eighteen who were seized. About Colinsburgh and the Largoward district it has been equally fatal, there having been in some families three or four deaths. And in the united villages of Elie and Earlsferry, about one-half of those seized have died. In this town and immediate neighbourhood, the mortality has likewise been great. Thus its destructive nature makes it a subject of keen interest, as scarlet fever has not heretofore been always considered a dangerous disease.

At first sight it may be doubted whether this disease is not the *cynanche maligna*, as this latter affection sometimes makes its appearance as an epidemic; but from all the instances that have come under my own observation, I am clearly of opinion that it is a different disease, and that for the following reasons:—

1*st*, In all the cases that I have seen, there have been no ulcers of a malignant kind on the fauces. In every instance, the ulcers have exhibited the appearance of small distinct white spots, which have not in any case run into one another.

2*dly*, I have observed some cases of this epidemic where the fever and eruptions were considerable, but where there was scarcely any affection of the throat whatever.

And 3*dly*, The slowness of its diffusion is contrary to what occurs in *cynanche maligna* when this latter disease appears as an epidemic. In this town and the districts I have referred to, the scarlet fever sometimes almost disappears for two or three weeks at a time, and afterwards bursts out afresh, attaking several families living contiguous to one another, almost at the same time.

From these considerations it is evident that this epidemic is the scarlatina as described by Dr. Sydenham and others; and its definition will perhaps be best given by describing some of the cases that occurred in my own practice. I shall only quote three or four of the worst cases out of about thirteen or fourteen that came under my observation, all the others being either somewhat similar, or comparatively mild, and terminating favourably.

On the 25th of February last, I was called to J. S., a fine boy, aged eight years, residing at a boarding-school, who had been quite well the day before. His teacher stated that, before going to bed the previous evening, he had complained of pain in the head and back, felt cold, and had a slight degree of shivering. He had his feet bathed in hot water, and was put to bed, taking at the same time a copious draught of warm gruel. When called next morning by the servant, it was found that he had been restless during the night, and now felt feverish, but was not so ill as to excite any uneasiness in the mind of his teacher, who about 7 o'clock gave him of his own accord a calomel powder. This operated pretty smartly as a purgative before 10 o'clock; and when I was called to him about 11 A. M., the fever had increased since the operation of the medicine. He now felt extremely hot; had great thirst; the face was flushed; he complained much of headach; and was exceedingly restless. He did not complain of his throat, but I could perceive by the thickness of his voice that the throat must be affected, and found upon looking into it considerable redness and swelling of the fauces. There was an eruption on the face, neck, breast, and arms; the pulse was 130.

℞. Tart. Antimonii gr. ii. solve in aq. ferventi. ℥iii. dein adde Acet. Scillæ ℥ss. Fiat mistura, cujus sumat cochleare minimum tertia quaque hora.

℞. Acidi. Nitrici gtts. xx. Aq. Fontanæ ℥viii. M. Fiat gargarisma.

At 6 o'clock P. M. the fever and restlessness had greatly increased. The eruption was now diffusely spread over the skin, appearing in some places as if above the cuticle; pulse 140. I applied the cold affusion, after which my patient felt much more comfortable. The pulse fell immediately to 116. At 11 o'clock P. M. it was again at 140; skin hot as ever. The cold affusion was again applied, and with the same good effect.

26th. This morning, in consequence of the fever being ardent, the cold affusion was applied at 6 o'clock, and also at 10. The patient felt cool and pretty well for a couple of hours or so after each application of the cold water. The pulse was lowered 30 or 35 beats per minute every time, but in the space of three or four hours it was as high as ever. I counted it to-day at 150. Complained more of headach; the throat was a good deal inflamed; skin perfectly dry; thirst not so great as might be expected.

Sanguinis emittantur de brachio ℥x.

℞. Calomelanos, Pul. Jalapæ *aa.* gr. iv. Pulv. Antimonial, gr. iii. M. Sumat statim. Cont. Sol. Tart. Antimonii et gargarisma ut antea.

This afternoon about 5 o'clock, notwithstanding the coldness of the weather, the patient called out most anxiously for the cold affusion, which was then applied, and again at 10 P. M. Always after the use of the bath the eruption appeared brighter than before. He had three stools of a dark colour since morning.

27th. The patient had passed a better night; skin softer to the touch, but still quite dry; pulse 130.

℞. Calomelanos, gr. vi.; Pulv. Opii, Pulv. Ipecacuanhæ, *aa* gr. i.; M. et in Pulveres iii. dividantur. Sumat unum quartis horis. Cont. Sol. Tart. Antimonii et gargarisma.

In the afternoon I observed numerous white spots on the fauces; but although the throat was worse, the other symptoms were abated; pulse 126.

℞. Acidi Muriatici, ʒss.; Syrupi Papaveris, ℥ii.; Infusi Rosæ, ℥vi. Fiat gargarisma.

28th. This morning the patient is much better in every respect; pulse 96; bowels rather confined.

℞. Sulph .Magnes. ʒvi.; Bitart. Potass. ʒi. Aq. Puræ, ℥viii. M. Sumat statim.

From this time he continued to improve. On the 1st of March he began the use of nitre and cinchona bark, with a moderate allowance of port wine. For some time his urine was scanty, and there were dropsical swellings of the skin in different parts. All these symptoms yielded in a short time to tonics and diuretics with an occasional purgative. About the 7th, glandular swellings behind the ears appeared, which gradually went off, by the use of the camphor liniment.

*Case* 2.—Feb. 27th. A young lady, aged 13, of spare habit, who was quite well yesterday, was this morning affected with sore throat and severe headach, attended with considerable fever; tongue covered with a thick fur; great oppression at the precordia; skin hot and rather moist. An emetic was given, which seemed to relieve the oppression, and in the afternoon the eruption made its appearance. At this time the throat was much inflamed, and she felt exceedingly restless. The eruption seemed as if completely under the cuticle. or rather the cuticle appeared like a diaphanous veil, covering the eruption and floating above it. At this time there was considerable diaphoresis, which was encouraged by a solution of tartarized antimony. An acidulated gargle was used every two or three hours, and four grains of calomel, with the same quantity of jalap, were given in the evening. The pulse was 130.

28th. The purgative operated freely: the headach was relieved, and throat rather better; the skin continued open; urine scanty and high-coloured; pulse 130. The diaphoretic, with the addition of small doses of nitrous ether; as also the gargle, were continued.

March 1st. Patient felt much better to-day, but the pulse continued still about 110.

℞. Calomelanos, Pulv. Jalap. *aa.* gr. iv. Pulv. Ipecacuanhæ, gr. i.; M. Sumit statim.

March 2d. The patient is much better in every respect to-day. Pulse 100. From this time she progressively improved, requiring no medicine except the cinchona bark with nitre, and once or twice a purgative.

*Case* 3.—Feb. 21st. A young woman, aged 15, of a florid complexion, had been complaining of headach and sore throat for two days, and to-day she has been much worse, being in a high state of fever, with great oppression at the precordia; tongue loaded; throat much inflamed; and an eruption all over the face, neck, breast, arms, &c. There was a great deal of mucus about the throat and fauces, and she had been retching, but could get nothing up; pulse 106.

℞. Pulv. Ipecacuanhæ, ℥ss. Sumat statim.

The emetic operated freely, and the violence of the retching produced considerable hæmorrhage from the nose.

22d. This patient is much better this morning; she slept pretty well after the hæmorrhage stopped last night, and feels comparatively well. The eruption seems to be going off, and the throat is much better; pulse 90.

℞. Calomelanos, gr. vi.; Pulv. Jalapæ, ℈i. M. Sumat statim.

23d. She was sitting up to-day, quite well to all appearance, and from this time progressively improved.

*Case* 4.—On the 16th of March, I was called to J. Boyd, a boy aged 4 years, who had had scarlet fever. The parents of the child said the case was so mild, that they had treated it themselves, giving the little boy occasionally a dose of salts and senna. It was now twelve days since he had been attacked, and for the last two days dropsical swellings of the skin had appeared in different parts of the body. The feet were at this time considerably swollen, as also the face and hands. If he lay on one side for two or three hours at a time, the cheek of that side became so much swollen as to close up the eye. He had hard glandular swellings behind the ears. His urine was scanty, thickish, and as black as tar. The stools were of the same odour and consistence. In fact, there could be no difference perceived between them. He did not complain of pain, except upon pressing the abdomen. The tongue was dry, and slightly furred; he had no desire for food; he did not complain of thirst; he was much emaciated; the pulse varied from 110 to 130.

℞. Calomelanos, gr. iii.; Pulv. Scammoniæ Compositæ, gr. v.; Pulv. Ipecacuanhæ, gr. ss. M. et fiat hujusmodi Pulv. ii. quarum unum nocte maneque sumend.

℞. Acet. Scillæ, ʒii.; Spt. Ætheris Nitrici, ℥i.; Syrupi aurantii, ℥ii. M. Cochleare unum minimum ter quaterve in die sumendus.

The patient was ordered chicken-broth, or beef-tea with toast, and any light nourishing food they could get him to take. The camphor liniment was used for the glandular swellings.

17th. There is to appearance no change since yesterday; urine as scanty as ever, and dark coloured. He has had two or three trifling evacuations of the same colour as before, and complains more of pain in his bowels; pulse 130.

℞. Sulph. Magnesiæ, ℥ss.; Bitart. Potassæ, ʒi. Aq. puræ, ℥viii. M. De quo capiat cyathum parvum omni hora donec alvus ter quaterve respondeat.

18th. The saline mixture produced several stools in the course of the night, and what has been passed this morning is of a thicker consistence. The urine is still small in quantity, but is not so dark coloured. It seems as if it were a mixture of healthy urine and tar water. Pulse 126. Cont. pulveres et mistura diuretica ut antea.

19th. The urine is more natural in appearance to-day; the feet and hands are not so much swelled; the face is much the same; little improvement otherwise. Pulse 120. Rept. mistura salina.

20th. He has had several stools since yesterday; urine still scanty, and very dark-coloured; pulse 130.

℞. Acetat. Potassæ, ʒii. Infusi quassiæ, ℥vi. Tincturæ Digitalis, ʒii. M. Cochleare unum mag. ter quaterve in die sumend.

21st. To-day this little patient appears better; he has voided a good deal of urine since last night; the swellings appear to be going off. From this time till the 25th, he continued to improve, having taken the diuretic mixture all the time, as also the cinchona bark with white wine, and an occasional purgative. His pulse, however, continued high, and varied from 100 to 126. On the 26th the fever increased, and the urine became more scanty; the face was more swelled than ever; the scrotum œdematous and shiny; pulse 140.

℞. Calomelanos, Pulv. Jalapæ, *aa.* gr. iv. Pulv. Ipecacuanhæ, gr. i. M. Sumat statim.

This was followed by a saline draught four hours afterwards. At night, although the medicine had operated, and seemed to give relief, yet by nine o'clock all the symptoms became worse. The scrotum was as large as two fists; skin dry and hot; pulse 150. Six ounces of blood were drawn from the arm; four grains of Dover's powder were directed to be given every fourth hour; and the scrotum and other dropsical swellings were ordered to be rubbed with camphor liniment. At this period the parents of the child had lost all hopes of his recovery, but were most anxious to do everything for him that was ordered. Although languid after the blood-letting he seemed easier. In half an hour afterwards the pulse was 140, and the heart's action seemed more free.

27th. A wonderful change for the better had taken place in this little patient since the previous night. He passed a tranquil night, and about seven o'clock was asking for food. His skin, although soft to the touch, was not moist; he had voided a considerable quantity of urine; the swelling of the face, hands, and feet had fallen. The scrotum was much the same; he had a stool this morning pretty natural in appearance; pulse 110. The blood taken last night exhibited a thick buffy coat on the top this morning; the crassamentum firm, cupped, and contracted to very small bulk, as compared with the quantity of serum, which was of a yellow colour.

The Dover's powders twice a-day were continued, and lemonade with supercarbonate of soda was ordered for drink.

28th. He is much better to-day; urine and stools pretty natural; scrotum much less in size; pulse 100. From this time he improved every day, and by the 1st of April was able to walk across the room.

The above little boy had a brother about two years old, who, when I was first called to them, was similarly affected with himself, but was not so much reduced in strength, neither had he so much fever. His urine and stools were as dark, however, and he had dropsical swellings of the skin in different parts. By the use of diuretics and purgatives he got well in a few days.

I have seen three or four other cases where dropsical swellings of the skin came on after slight attacks of scarlet fever, attended also with fever, but they yielded easily to cinchona bark in combination with nitre, given along with these occasional purgatives.

A difference of opinion has prevailed with respect to the treatment of this kind of dropsy following scarlatina, some recommending blood-letting, while others prescribe tonics. In a case like that of the boy Boyd, when all the symptoms yielded to the diuretics and purgatives, and when it is considered that he was twice convalescent, it is difficult to say whether earlier blood-letting might have been of advantage. He had no pain in any part of his body; no inflammatory symptoms further than the increased arterial action; his brother's urine was nearly as dark-coloured as his own, and they had both dropsical swellings. The chief difference between the two cases was, that the eldest boy had from the first a much greater degree of fever than the other. Where there is much arterial action in connection with the œdema, and when this continues after the other symptoms have abated, I believe it will be safe and beneficial to abstract blood; and further, if the dropsy does not yield to other means, I would advise blood-letting in every case where the fever runs high.

In the first case noticed in this paper, that of J. S., we have a striking proof of the benefit to be derived from the cold affusion. Every time it was applied it lowered the pulse about 30 in the minute, and the patient was comparatively easy for a couple of hours afterwards. I saw another case where the same effect was produced; but great caution should be observed in the use of this remedy. If, for example, it were employed where there is any diaphoresis, and where the eruption does not come freely out, it would in my opinion send it in, and produce much mischief. It is a powerful auxiliary when skilfully used, but would become a dangerous enemy if improperly employed.

It appears to me that in every stage of scarlatina, the frequent use of acidulated gargles is of the greatest consequence, and that, until after their use, purgatives should in no case be administered. The disease originates in the throat and fauces; it is, therefore, evident that, if the morbific secretion is carried from thence into the alimentary canal, it will be diffused through the circulating system, and thus add greatly to the violence of the attack. The constant use of gargles has this advantage also, that it prevents any irritating matter from lodging in the ulcers, and thereby causing their extension and consequent sloughing.

I have not seen any of the cases that have terminated fatally, otherwise I would have inquired into the morbid appearances after death.

*Edinburgh Med. and Surg. Journ.*

---

*Some account of the Tubercular form of Elephantiasis, as it presents itself in the Island of Ceylon.* By Thomas B. Peacock, House-Surgeon of the Chester Infirmary.—During two visits which I have paid to Ceylon I have each time inspected the hospital for the reception of the cases of elephantiasis, first in company with Dr. Young of the Ceylon Rifles, and on the second occasion, by the kindness of my friend Dr. Kinnis, alone, in order that I might be able to devote more time to the observation of the cases than can generally be afforded when in company. It is the result of these visits which I purpose to embody in this paper.

The Leper Hospital is situated on the northern bank of the Kalany, opposite to the village of Mootwall, and distant about four miles from Colombo. It commands a fine view of the river, which here is about a quarter of a mile broad—bordered by cocoa nut gardens, and broken by numerous small jungle-covered islands, with the anchorage of Colombo in the distance.

The building consists of a centre, surrounded by an open verandah, the residence of the medical sub-assistant having charge of it, and two long wings devoted to the reception of the patients. To them the entrance is through an old-fashioned gate, surmounted by the arms of the Dutch East India Company, from which two small doors communicate respectively with the men's and women's side of the establishment. There are two square plots of grass, having the wards built on one side, while the other opens into an extensive garden. The wards are lofty and of one story, neatly whitewashed, and paved with brick.

In speaking of elephantiasis it is necessary that I should explain, that it is to the tubercular form of the disease, or that described by most authors under the name of elephantiasis of the Greeks, or more popularly of Leontiasis, to which my remarks are intended to apply—a disease very distinct from the Barbadoes Leg, the *Bucnemia Tropica* of Good, or the *Elephantiasis Arabum* of other authors, though both prevail in the same localities, and are often, unless I am mistaken, found coexistent. The cases, then, of tubercular elephantiasis of which I possess notes are twelve in number—these forming the most remarkable examples of the disease in the hospital at the time of my visits. Its inmates, in all forty-five, being composed by no means entirely of these cases, but including eight insane patients; two labouring under phagedenic sores, and several other cases of disease. Of the twelve referred to all were males; one had been in the hospital since 1802, and another, who had also been long there, died in the interval between my first and second visit. Their ages ranged from 25 to upwards of 50; one had suffered under the disease for upwards of 40 years, another 30, and others 10, 9, 8, 7, and one only 5 years. Dr. Ainslie has stated that the disease generally begins between the ages of 20 and 30, and rarely after 40. Those whom I examined were first attacked by it at the ages of 30, 22, and 19, two at 17, one at 16, and one at 14; yet, as my inquiries were carried on through the medium of an interpreter, these dates cannot be altogether depended upon. One of the men was descended from Creole parents, and two were the sons of European fathers by native women; the rest were of the lower class of Singhalese; several had served as soldiers in the native corps.

From the histories given me of the mode of attack and progress of the disease in their several cases, it would appear that the limbs are generally first affected, more especially the small toes. It usually commences as a small pimple, differing little in colour from the parts around. Several, however, said it was whitish, and some that it felt hard. The patch loses its sensibility, and becomes covered with a furfuraceous scale, which, separating, leaves underneath it a point of spreading ulceration, secreting a kind of ichor. The disease having thus commenced, proceeds to a greater or less extent, and then heals, leaving behind it a hard, thick, and irregular cicatrix. In some cases it would appear as if the diseased action had there exhausted itself, the part undergoing no further change, while it would reappear after a variable interval elsewhere; a course which was stated to have generally been followed where it first manifested itself in some other part than the face or extremities. In other cases, after having remained healed for months, perhaps years, the ulceration would return in the same situation with renewed energy. As the disease advances the adjacent parts become hard, lobulated, and fissured; sensibility is extensively impaired; and numerous hard tubercles with ulcerated apices make their appearance. In a while the ulceration implicates the joints; the joints become denuded and separate, till, by a continued process of healing and spreading, the victim gradually sinks under the load of disease. Long, however, before it has attained this extent, the distant parts have assumed the same action. If the extremities have been first attacked, the tubercles have begun to develope themselves on the face; if the latter organ had first fallen under the disease, the extremities have already suffered from it.

In its more advanced stages the deformity occasioned is often most frightful. In the face the *alæ nasi* fall in; the septum gives way; the extremity of the nose becomes a mass of tubercles; the lips are tumid and knotty; the lobes of the ears enlarged and fissured; and the eyebrows, thickened and frowning, are studded with small nodes. Nor do the mucous membranes escape. The tongue and inside of the cheeks become thickened and lobulated, and the nostrils discharge a fœtid secretion in a still worse state. The features are altogether distorted. The eyelids, tumid and irregular, become useless as a protection to the globe, and, pressing on the conjunctiva, cause it to inflame and ulcerate. The cornea becomes opaque, or, the parts sloughing, the organ collapses. Several had lost their sight from this cause; and one presented the most aggravated example of staphyloma I have seen.

In the limbs the ulceration removes the phalanges of the fingers and toes. Almost all the persons I examined had lost one or more of the small bones; and the man who had so long been a patient in the hospital had not, I think, one toe or finger which had not suffered in this way. In some the tarsal or carpal bones are affected; and in one, in which the general form of the foot was retained, and the bone of but one toe had been lost, the tarsal bones had ulcerated their way out, and left the instep soft and flexible in all directions. Much difference has existed amongst authors as to the existence of the glandular swellings in these cases. In one man, where the chief seat of the disease was in the face, the submaxillary and cervical glands were hard and swollen. And in another, where the extremities were chiefly affected, tumours existed in each groin, that on the right side being the largest, and ulcerated at several points. Both were extremely hard. No bands of absorbents could be detected. In the early stage I mentioned the impairment of the sensibility in the part affected. As the disease advances, however, the loss of sensation becomes entire; the patient will bear to be pricked with a sharp instrument without any complaint of pain; and in one person the conjunctiva on the eyeball was rendered perfectly insensible. In regard to this symptom we also find very contradictory statements amongst writers. Robinson has described two varieties of the disease, one of which is characterized by its presence, while in the other it is absent. Dr. Ainslie, on the contrary, states, that he has never seen any case in which it has not been combined with the other symptoms; a statement which completely corresponds with my observations, though in some instances the degree to which it existed was much greater than in others. Once or twice I noticed it attended with partial paralysis of the motor power, as the tongue being drawn to one side, or the dropping of one arm.

While the local disease is committing the ravages I have described, the patient rapidly loses flesh, and the appetite to supply the deficiency becomes voracious. Seldom have I seen emaciation to an equal degree in any disease of this climate. Indeed, as some of the miserable victims are seen lying under the shade of the verandahs to gasp a little pure air instead of the foul atmosphere of their wards, with their limbs drawn up to their bodies, and their whole frame dwindled into the smallest space; it required some effort to believe that the wretched form was still possessed of life; yet so keenly do they cling to existence, that several times did I see nails springing from the second, nay even from the first phalanx of their fingers, where the others had respectively fallen by the extension of the malady. With regard to the state of the sexual power, writers have given accounts as contradictory as on some other parts of the pathology of the disease. On the one hand, we are informed that it becomes greatly impaired or entirely lost; on the other, that it exists to an unusual degree. Here, too, I must concur in the report of Dr. Ainslie, who describes the loss of power and wasting of the testicles as of invariable occurrence in the latter stages,—a remark fully borne out by my observations at the Leper Hospital of Colombo. In some cases those glands could scarcely be detected, and there was an almost entire absence of hair from the chin and pubes; and in the young man, then 25 years of age, who had been attacked with the disease before he was 14, the signs of puberty had not developed themselves.

In other cases, however, where the disease had been of much shorter duration, the attendant gave me to understand that the sexual feeling was certainly not impaired.

It would not be expected that, where so frightful a malady is preying upon the constitution, the mind should maintain itself unaffected amidst the ruin. We find, indeed, that, early in the progress of the disease, its powers begin to decline; in the early stage its victims are suspicious, and a burden to themselves. They are continually involved in quarrels with each other, and their attendants, while in the latter periods of the malady they sink into the feebleness of a second childhood, or into a gloomy fatuity.

The object which I have planned for myself in this paper, of recording only what came under my own observation, will force me to pass very cursorily over two most interesting portions of the pathology of the disease. I refer to the symptoms ushering in the first appearance of the malady,—a period of the more importance in the circumstance, that then only will attempts at treatment probably be of any avail; and to the morbid appearances observed in the bodies of those in whom it proved fatal in regard to the first.

I find on referring to my notes, that in only two cases did I elicit any information on the former point, and these only under the obscure name of fever; indeed, when we recollect the length of time which had elapsed since the occurrence of the malady, it is obvious that any account furnished by the patients themselves of this stage, must have been most uncertain.

On the morbid appearances found after death, my information is still more defective. The patients dying of this disease in the Colombo hospital, have not, I believe, been generally examined; and Dr. Kinnis, whose paper on the disease as it fell under his observation at Mauritius, published in a previous volume of this Journal, is the best I have seen, and leaves little to add, informs me that the persons labouring under the severe forms of the malady are sent thence to the Island of Diego Garcia, thus preventing his investigating this portion of its pathology.

Having now given a hasty sketch of the disease, and endeavoured to describe the forms which it generally assumes, for indeed its phases are as various as are its individual examples, it remains for me to offer a few remarks on the causes to which it is to be ascribed. On turning over the pages of the different authors on this subject, their statements are found to be so varied and often so contradictory, that it is difficult to know which party to follow. Some have asserted that it is contagious, and most that it is hereditary, or at least that it is more likely to appear in children born after their parents have suffered from the disease than in others. Good regards these facts as so well established, that he has introduced them into his definition of the disease, and states that the Hindu physicians concur in the belief of its contagion. I should feel unwilling, on the strength of a few days observation, to contradict the reports of those, who have had much better opportunities of investigation than myself; but, if I mistake not, the general answer to my inquiries in Ceylon was, that it was not there regarded as contagious, nor did I hear an instance of any attendants at the hospital having been attacked by it. With respect to its hereditary tendency, all but one of the twelve whom I examined, denied that any of their relations had suffered from the disease, and in his case that relation was an uncle. I am, therefore, inclined to doubt whether these causes operate to the extent which has been supposed, and to inquire how far the common sources of disease are equal to the production of so frightful and intractable a malady.

The first feature which presents itself in this investigation is the influence of heat and humidity. I am not aware that the disease ever presents itself except within or near the tropics, where the temperature is high and the transitions rapid. In Ceylon, the west coast is throughout only elevated a few feet above the sea; the rivers are almost closed at their mouths by high banks of sand, and form extensive basins, with low marshy banks, teeming with vegetation. During the rainy season the rain falls in torrents, with short intervals, in which the clouds roll off, and the sky is left murky and thick; the rivers, incapable of quickly emptying themselves, overflow their banks, and leave extensive marshes to dry on the return of fine weather. The temperature ranges from 80 to 82 and 84 at noon, while at night it often falls to 60 with great feeling of cold. We have here, therefore, a most fruitful source of malaria; and if to Europeans, possessed of every protection against disease, the climate be healthier than that of almost any other portion of the tropics, we cannot be surprised that the natives, living as they do in wretched hovels covered with cocoa nut leaves; devoid of almost all clothing; supporting life on an innutritious diet of rice and plantains, and in many instances indulging to excess in spirits, should fall into a cachectic state of system, forming a nidus, in which a disease, more hideous than any known in the more favoured regions of the globe, should develope itself.

High temperature and sudden changes are not, however, alone sufficient to give rise to the disease, since I am not aware that it is common in the interior either of India or of Ceylon. At least, during three weeks which I spent in the Kandyan territory, I do not recollect to have seen a single instance of it; yet amongst the mountains of Ceylon, the transitions are both greater and more rapid; thus at Newera Ellya, a convalescent station 6000 feet above the level of the sea, where I spent ten days, we had, with a nearly vertical sun, a temperature of 76° to 78° at mid-day, falling rapidly at sunset, and being 35°, 32°, and 30° at sunrise; yet the diseases from which the natives suffer are entirely inflammatory affections of the chest, and the European troops wear the healthy hue of men fresh from home.

High temperature, therefore, and rapid transitions will not give rise to the disease. The determining influence I am inclined to regard as rather of a moral nature. The coast district of Ceylon has for ages been under foreign dominion; latterly the Portuguese, Dutch, and British have severally held it in subjection. Its inhabitants must be regarded as a general mixture of races; and when we consider the low social and moral condition of a people so constituted, in which the just relations of husband and wife, of parent and child, are almost unknown, we have, I think, an ample source for the development of the evil. It is to the absence of this influence, I conceive, that the Kandyan inhabiting the mountain district of Ceylon,—clothed and housed scarcely better than the native of the coast district,—living on food as innutritious, and exposed to transition of temperature still greater, yet possessing many of the virtues of a mountain race, active, temperate, and industrious, is free from the scourge to which his feebler and more degenerate neighbor too frequently falls a victim.

In conclusion, I must as briefly refer to the treatment of this complaint as I have before necessarily done to several other of its features. The Leper Hospital at Colombo seems to be chiefly intended as an asylum for the indigent and diseased. The insane, the distinctly syphilitic, persons with aggravated forms of the Barbadoes leg, and those labouring under elephantiasis, are alike lodged within its wards, though without hopes of their being benefited by treatment. If, indeed, in the latter disease, medical means were ever likely to be of any avail, I am inclined to think, it would only be at the time of the invasion of the symptoms, or in the variable interval which elapses between their first attack and recurrence. At this period, however, they take only some feeble herbal medicines, such as are generally prescribed by the native doctors, and only come under the care of European medical men, when the disease is fully established, and when it is, I fear, ever likely to form one of the *opprobria medicinæ.*—*Edinb. Med. and Surg. Jour.*

---

*On the Necessity of studying rare Cases, being the introductory lecture to a course of Physiology.* By M. Lordat.*—When the old University of Paris was suppressed, and the republican government substituted the *Ecole de Santé* for the *Faculté de Médecine*, the director, besides his official duties, was appointed to explain the doctrines of Hippocrates, and the history of rare cases; that is to say, the history of the extraordinary phenomena which have been observed at various periods in some individuals, whether belonging to anatomy or physiology. It appears, however, that he did not give a single lecture on the latter subject; and in the catalogue of professors in the Parisian faculty of medicine, we no longer find one whose office it is to explain rare cases. I am surprised that this part of the study of physic should have fallen into oblivion, indifference, or discredit.

On examining the question more nearly, however, it seems to me that all parts of the history of rare cases are not equally neglected; cases belonging to anatomy are studied, but those appertaining to physiology are passed over. Monstrosities, anomalies in the distribution of the blood-vessels, club-feet, imperforations, and remarkable pathological phenomena belonging to surgery, are carefully collected. If they are not the subject of a special lectureship, they do not lack celebrity; they re-echo in the academies; they are described at length and commented on in the journals; and they have been long mentioned in lectures on surgery and anatomy, with which they have more or less connection. But it is not so with

* Abridged from the Gazette Medicale of July 18, 1840.

the singularities observed in the exercise of the vital force of man, without material alteration. The facts formerly taught in the schools, and published under the names of *casus rariores*, *historia medica admiranda*, or *praxis miranda*, are now unknown; and those which are still presented to us by nature are unnoted, disdained, and rejected with derision.

Why are two kinds of facts, which are equally singular, treated so differently? We see, on reflection, that they are derived from the same source; that congenital deformities or monstrosities are the effects of the same cause which produces the most singular transitory phenomena, namely, the variations which happen in human *dynamism*. How is it possible to think so much of one kind, and so entirely to undervalue the other?

The immediate or proximate cause of this general disposition is to be found in the preference given by the majority to material knowledge over intellectual notions. The presenee of a permanent fact which strikes the senses, absorbs the whole attention: its origin and cause are forgotten, while the observer is occupied in classing it, or employing it for some mechanical purpose. But a vital, fugitive, and extraordinary phenomenon is often noted by the skilful alone; it vanishes before a sufficient number of witnesses have been able to satisfy themselves of its reality, and make it sufficiently notorious. Since nothing visible remains of it, we can only investigate its relations, affinities, and causes; an intellectual labour which is very difficult, and is no longer the mode of the day.

The disregard of this study proceeds from the prevailing error of neglecting the theory of physic, and believing that the causes which we have seen are sufficient to form a complete nosology. The majority of physicians have forgotten the necessary truth, that the number of things which the most busy practitioner has seen is infinitely smaller than the number of those which he has not seen. From this forgetfulness proceeds the disuse of many doctrinal principles of a high order, the ignorance of many vital facts necessary to be known, contempt for the past in medicine, profound indifference for anthropologic erudition, a great desire to appear well informed in rare cases of the anatomical kind, and an extreme wish to show oneself an *esprit fort* (incredulous) with respect to these singular phenomena in the domain of physiology, which we have not witnessed ourselves. Nay more, we now actually see men, otherwise estimable, who, after applying the test of their senses, quite at their ease, to physiological phenomena differing from those with which they are familiar, seriously repeat what Fontenelle uttered as an epigram: "*Je l'ai vu, et je ne le crois pas.*"

This last caprice, which has been seen sporadically in all ages, from the effect of individual ignorance, and which has now become epidemic, from the fault in teaching which I have mentioned, might be injurious to your progress if you were affected by it.

You do not suppose, I hope, that I declare myself the general champion of all the writers of wonders, of all the historians who have accumulated marvellous narratives, such as Herodotus, Pliny and Livy. What I wish is, that you should distinguish judicious and observing physicians from other narrators; that you should not be armed with prejudices against extraordinary facts, either when it depends on you to verify them, or when you are able to calculate on their credibility, whether from their relations to the knowledge you have of human nature, or from the rules of evidence.

I have endeavored to find out what are the accusations by which the enemies of rare or new cases decry their study; but I have listened in vain, and have heard nothing but repugnance, obstinate and unreasoning incredulity, and indirect arguments; but never what the logicians call an argument *ad judicium.*

The following is what the incredulous allege:—

1. That novel cases of the physiological kind are unworthy of confidence, and consequently do not deserve any attention.

2. That rare cases, in general, are useless in the practice of physic, for it is an axiom that *rara non sunt artis.*

3. That they have been collected to entertain, and not to instruct; they are æsthetic, and not philosophical.

4. The physician cannot employ them, as the public does not believe them, and even laughs at them.

1. *Novel cases of a physiological kind are unworthy of attention.*

When I wished to analyse this important assertion, and reduce it to clearer terms, some of these cases were declared to be impossible, others incontrovertibly uncertain, and others anomalies.

But when I asked for a demonstration *à priori* of the impossibility of the most extraordinary cases, I did not find a man capable of giving it. Those who consider the rare cases narrated in the writings of physicians as absurdities, are excellent *savans*, naturalists, or mathematicians; but they are unversed in the knowledge of human dynamism, and, conseqently, unable to conceive the extent and limits of this power; so that they employ the word absurdity without reflecting on its meaning, and with the exaggeration of our men of fashion and pretty women.

Do not misuse this term, or confound the impossible with the incredible. The impossible is an absolute and demonstrable thing. The incredible depends on a relative mental disposition, which differs in each individual, according to the quality and number of the ideas which he possesses concerning the thing in question. This disposition is not composed merely of the distance which separates the fact examined from the knowledge with which the mind is furnished, but it also depends on the probability of the testimony by which the fact is accompanied.—*Lond. Med. Gaz.*

---

*Strychnine in Neuralgia.* By J. Pidduck.—In the administration of active remedies, it has been, I conceive, too much the custom gradually to augment the dose to the utmost limit of tolerance. This mode of procedure is more calculated to exhibit the poisonous than to elicit the medicinal effect of medicines. It is a mode of trying the powers of resistance on the part of the patient, instead of proving the efficacy of the remedy.

The question in every case is, not how large a dose the patient can take with impunity, but, by how small a dose his disease can be cured; for it will not, I think, be denied, that every medicinal agent which is potent for good, is equally potent for evil, when unduly, as well as when untimely administered.

This a medical canon of universal application. The paramount duty of the prescribers, therefore, is carefully to note the operation of the remedy upon the disease, and if the disease be yielding to the influence of the remedy, although it may be slowly, instead of augmenting the dose to the utmost limit of safety, to suspend the remedy, from time to time, in order to ascertain what progress has been made towards the cure.

This rule applies to the administration of all energetic medicinal agents—to mercury in the cure of syphilis; to tartarized antimony in pneumonia; to calomel and opium in pleurisy; to acetate of lead and opium in hæmorrhage; to quinine in intermittent fevers; to colchicum in gout and rheumatism; to conium in carcinoma; to digitalis in dropsy; to iodine in bronchocele; to opium in tetanus and other spasmodic diseases; to nitrate of silver in epilepsy; to strychnia in paralysis. In each of these cases, experience has shown that results far more satisfactory have been obtained from these active agents in medicinal than in poisonous doses. Inattention to this simple rule has doubtless been frequently the cause of their failure.

With these few prefatory remarks, I proceed to the immediate subject of this paper.

The attention of the profession has been for nearly thirty years directed to the nux vomica, in the form of its alcoholic extract, as a remedy in paralysis; and for nearly twenty years to its alkaloid, strychnia, for the same purpose. To Dr. J. L. Bardsley we are indebted for several valuable cases, in which the efficacy of strychnia in paralysis was fairly treated.* It is to the utility of strychnia in another class of diseases—the various forms of *neuralgia*—to which I desire to invite attention. Mine was first directed to the employment of strychnia in a case of this description, from conceiving that neuralgia might be occasioned by a diseased condition of the nerves of sensation, similar to that which occasions one form of paralysis in the nerves of motion, viz., an injected state of the capillary vessels of the investing membranes of the nerves. That the loss of voluntary power in the one case, and the acute pain in the other, proceed from one and the same pathological condition—an alteration in the relative state, or a deviation from the normal relation, which ought to subsist between the capillary vessels and the nervous fibrils.

It is probable this injected state of the capillaries may be owing to the altered qualities of the circulating fluids, which occasion the loss of tone in those minute vessels; and hence their admission of the red particles of blood. The first case in which I tried the strychnia was in that of a maid-servant, who suffered from neuralgia of the infra-orbital branch of the fifth pair. The agony was dreadful. After a variety of means had been employed without any alleviation, strychnia, in the dose of a twelfth of a grain, mixed with sugar was prescribed. After the third dose relief was obtained. The pain entirely ceased before a grain had been taken. In a few days the pain returned in a slighter degree; the medicine was resumed with the same good effect, and there has been no relapse.

The second case was of another maid-servant. The affected nerve was the infra-orbital branch of the fifth pair, the pain extending to the joint of the lower jaw, the motion of which, in eating, speaking, &c., brought on paroxysms. After a few doses of the twelfth of a grain of strychnia, the pain ceased, and has not returned.

The third case was of sciatica in a labouring man. One grain of strychnia, divided into twelve doses, sufficed for the cure. The pain returned after several weeks. The cure a second time was effected by the same remedy, and he remains free from pain.

The fourth case was a complication of neuralgia, and loss of power in the right hand and arm, in a female teacher in an infant school. On attempting to write, paroxysms of severe pain, and loss of control over the muscles, compelled her to desist.

After taking the twelfth of a grain of strychnia three times a day, for three days, a great increase of pain in the arm and hand supervened, with violent agitation affecting the whole side. Relinquishing the medicine for a few days, the pain and agitation subsided, and she felt the arm much stronger and less painful. The medicine was resumed again, and relinquished on a recurrence of the same symptoms in a slighter degree. A third course of the medicine effected a cure. The improvement in

---

* Hospital Facts and Observations.

the state of the general health was no less striking in this case than in the hand and arm. To use her own expression, "She had never taken any medicince from which she had derived so much benefit as from those pills."

The fifth was a case of sciatica in an elderly woman. The pain had existed for several months, and at times it was excruciating: it commenced with lumbago. Tenderness on pressure being felt in the sacral nerves, a few leeches were applied, and blue pill at night, followed by castor oil in the morning, was prescribed. By these means the tenderness at the lower part of the back was removed, but without any alleviation of the pain along the course of the sciatic nerve. Strychnia, in the twelfth of a grain dose, was given; three grains of the medicine, taken with occasional intervals of a few days, effected the cure, with a very decided amendment in the state of her health.

The sixth case was severe neuralgia of the left arm, accompanied with cerebral symptoms, fits of vertigo, and loss of recollection. The patient had formerly suffered from epileptic attacks, and had obtained immunity from epilepsy by the use of strychnia, in poisonous doses. The dreadful agitation she had experienced under the influence of this medicine rendered her unwilling to have recourse to it again. After a few doses of alterative and aperient medicine, she commenced with the small dose of strychnia. The same symptoms, only in a very moderate degree, followed the use of the remedy—unsteadiness in walking, and irregular twitching in the muscles, with aggravation of pain in the arm. The strychnia being omitted, the pain subsided, and so on several times, each time the pain being less, till it has almost entirely ceased. The removal of the cerebral symptoms preceded the diminution of the pain in the arm, and an amended state of her general health has become apparent under the influence of the strychnia, which she is still taking.

These six cases are insufficient to establish the efficacy of the strychnia in neuralgia; but the result has shown the power of the remedy over painful, no less than over paralytic, affections of the nerves; and it has been so far satisfactory as to entitle the strychnia, in minute doses, to a more extensive trial in this most distressing class of diseases. As in cases of paralysis strychnia has only been found of use where there was no organic lesion of the cerebro-spinal centres, so, in cases of neuralgia, no hope of benefit from strychnia is to be entertained, where there is any mechanical cause of irritation affecting the nerve at its origin or along its course. The difficulty of ascertaining the existence of such a mechanical cause oftentimes is confessedly very great. The only dietetic restrictions are careful abstinence from any thing sour,—vinegar, cider, acescent wines, unripe and acid fruits, common salt in large quantity, and saline medicines.

The *modus operandi* of strychnia, besides its direct influence upon the nervous system, seems to be similar to that of all bitter tonics—increasing the appetite for food, promoting digestion, and as a consequence occasioning constipation. But this is perhaps not to be regarded as a morbid condition, but as the effect of a more perfect digestion, and the absorption of a larger proportion of nutriment into the system.

It may be owing to the introduction of a larger quantity of perfectly assimilated chyle into the blood, that harmony is restored between the circulating fluids and the nervous system, and hence the benefit of the *diète blanche* in hysterical and other nervous disorders.—*London Medical Gazette.*

---

*Medical Experiments.* By Dr. J. C. G. Jörg.

(Continued from p. 634.)

*Camphor.*—This drug was first taken dissolved in spirit, and afterwards in substance. The solution contained a grain in eight drops, and the doses were from 4 to 28 drops. When taken in substance, the doses varied from half a grain to 12 grains. Dr. Jörg likewise gives at length Alexander's famous case, where that bold experimenter nearly destroyed himself with ℈ij. of camphor. The reader will find a short abstract of it in Christison, p. 647. It appears from the experiments of Dr. Jörg, that camphor is primarily a stimulus to the intestinal canal and brain, and secondarily excites the urinary and genital organs, and the vascular system. It affects the intestinal canal chiefly as a nervine remedy, not so much by any bitter or acrid matter, as by a volatile ingredient; hence it acts almost like alcohol or any powerful and purely spirituous fluid; still these are not its only effects, for its bitter and acrid constituents also come into play. Its power of acting like a spirituous fluid, and causing a sense of burning in the mouth, and of warmth in the stomach and intestines, is increased by taking it dissolved in spirit; in this way, too, its nervine properties become more prominent.

Camphor is to be given in atonic diseases only; and it is not a sufficient indication for its use that some function is suppressed which it has the power of stimulating, unless the *vis vitæ* is at the same time in a low state. Thus it would be wrong to give camphor as a sudorific in acute fevers with a dry skin. From its property of stimulating the intestinal system, it is admirably adapted for diseases where, with great general debility, the functions of the intestinal canal are especially paralyzed. Since camphor does not inflate the abdomen, as valerian, serpentaria, and arnica do, it is unquestionably to be preferred in intestinal tympanitis of an atonic or putrid, but not an inflammatory character."

The dose should not exceed half a grain to a grain every four or six hours.—*Ib.*

